# A Standardized Protocol for Assuring the Validity of Proteomics Results from Liquid Chromatography–High-Resolution Mass Spectrometry

**DOI:** 10.3390/ijms24076129

**Published:** 2023-03-24

**Authors:** Andrzej Gawor, Ewa Bulska

**Affiliations:** Biological and Chemical Research Centre, Faculty of Chemistry, University of Warsaw, Żwirki i Wigury 101, 02-089 Warsaw, Poland; agawor@chem.uw.edu.pl

**Keywords:** quality control, valid result, mass spectrometry, proteomics, ISO/IEC 17025:2017, ISO 15189:2022

## Abstract

Significant advances in the technological development of mass spectrometry in the field of proteomics and the generation of extremely large amounts of data require a very critical approach to assure the validity of results. Commonly used procedures involved liquid chromatography followed by high-resolution mass spectrometry measurements. Proteomics analysis is used in many fields including the investigation of the metabolism of biologically active substances in organisms. Thus, there is a need to care about the validity of the obtained results. In this work, we proposed a standardized protocol for proteomic analysis using liquid chromatography–high-resolution mass spectrometry, which covers all of these analytical steps to ensure the validity of the results. For this purpose, we explored the requirements of the ISO/IEC 17025:2017 standard as a reference document for quality control in biochemistry research-based mass spectrometry.

## 1. Introduction

The reliability and validity of analytical results are the most important and fundamental features in the evaluation of any measurement system [1], particularly in the biochemistry area. They increase transparency and decrease opportunities to insert researcher’s bias in qualitative research. The strategy of investing in the development of new technologies requires conducting basic research, implementing new concepts in the field of economy or medicine (often based on mass spectrometry results), monitoring the practical effects of this research, and exploring the possibility of new applications. This research can be carried out in scientific laboratories (both in the commercial sector and at universities), R&D laboratories, and laboratories accredited according to international quality standards. Obtaining accreditation from an accreditation body is the demonstration of the highest competence in the field in which the laboratory is specialized. However, due to the nature of the research, it is impossible or often challenging for a laboratory that is involved in nonroutine research to obtain accreditation. The development of new technologies is a source of improvement in the quality of human life and the well-being of societies, and the main engine of the modern economy. Decisions are constantly being made globally on the results of measurements, assuming their reliability and validity. Comparison of the results obtained, particularly in scientific, medical, or product safety research, requires laboratories to operate in an identical and reproducible manner. All efforts to ensure the validity of measurement results, regardless of whether they concern simple chemical measurements such as pH measurement or advanced instrumentation methods, are extremely important. However, it is widely argued that nonroutine work does not fit easily into a highly documented and formalized quality system. For this reason, laboratories are trying to implement various approaches to ensure the validity of results based on their experience in the field, technical documentation of equipment, and good laboratory practices rather than compliance with formal standards. However, there is still a lack of standardized operating protocol in many areas of advanced analysis, in particular in proteomic analysis.

Proteomics is one of the ‘omics’ sciences that has developed rapidly, especially in the field of biochemistry, as it provides beneficial information on the identification, expression levels, and modification of proteins. In a wider meaning, proteomics is the study of the interactions, function, composition, and structures of proteins and their cellular activities [2]. Mass spectrometry-based proteomics has contributed significantly to the detection of drug target identification [3], and alteration of expression patterns in response to different environmental factors such as diet enriched in selenium compounds [4,5] or fluorinated drugs administration [6,7]. Modern proteomics methods have also recently become useful in neurodegenerative diseases in developing methods for the analysis [8] and identification of new biomarkers for early diagnosis and disease progression [9,10,11,12]. Many proteomics studies are also being conducted to identify novel proteins and biological functions in plants [13,14] and on model organisms such as *Tetrahymena thermophila* to elucidate the involvement of many key proteins in the basis of biological processes [15]. There are several reports in the literature on the use of proteomic methods in antidoping tests [16], early medical diagnosis [17,18,19,20], and candidates for vaccine production [21].

Proteomic analysis, using high-resolution mass spectrometry, is a multistage process involving: (i) sample preparation for measurement; (ii) chromatographic separation of a mixture of proteins or peptides and analysis of the specific components of the sample using an appropriate MS analyzer; and (iii) interpretation of the results obtained using relevant databases and bioinformatic software tools. The advanced techniques based on high-resolution mass spectrometry are characterized by high complexity, and mass spectrometry-based proteomics experiments might be affected by the variation in individual samples in a measurement series over time, especially in quantitative methods [5,6,22,23,24,25,26], which is a limitation in obtaining accurate and reproducible results. Papers on proteomic analysis often omit a clear description of how the quality of the results was ensured and how their validity was evaluated. Although in many publications the authors pay attention to the optimization of measurement parameters, and they are certainly aware of the need to ensure the validity of the obtained results, they are certainly using different approaches. Considering various approaches, we concluded that it is worth using commonly accepted documents that clearly show a straightforward way of proceeding.

Thus, compliance with the general guideline provided by an international standard: ISO/IEC 17025:2017 “General requirements for the competence of testing and calibration laboratories” [27] can be very beneficial for quality control of any analytical procedure, including proteomics. Furthermore, the requirements of the revised standard for medical laboratories: ISO 15189:2022 “Medical laboratories—Requirements for quality and competence” [28], which includes also the requirements of the ISO/IEC 17025, can also be useful in this field, particularly in the context of medical diagnostic studies using mass spectrometry techniques. Unfortunately, the application of both standards to design the quality control protocol of the measurements by high-resolution mass spectrometry (HRMS) is not a widespread activity, and, consequently, there are only a few relevant studies [29,30,31].

Having in mind that the entire analytical system in the proteomic analysis consists of two modules with different but fully coupled physicochemical bases, the metrological approach should include both. This means that the quality control approach should cover both chromatographic parameters as well as mass spectrometry parameters.

In respect of the chromatographic parameters, the reproducibility of the separation processes is the most important requisite for a reliable comparison among different runs and for ultimately obtaining quantitative information about the proteome under analysis [32,33,34]. Before analysis, it is important to check and examine the experimental conditions of chromatography and the stability of selected parameters such as peak widths, peak shapes, and retention time. The frequency of the calibration of liquid chromatography coupled with tandem mass spectrometry (LC–MS/MS) depends on the required mass accuracy. Calibrants are required to adjust the mass range of any coupled measuring system, it is crucial to use calibration solutions compatible with the ion source. In proteomic analysis, the most commonly used ion source is the electrospray ionization (ESI) system. To check the stability of chromatography parameters in proteomic analysis, a simple calibration mixture containing low levels of peptides, for instance, received after enzymatic hydrolysis of a single protein, is used [35]. The low complexity of this quality control solution is essential. During the ionization process in the ion source, peptides create many pseudomolecular ions with multiple charges [36].

It is commonly accepted, that Section 7.7 “Ensuring the validity of results” of the ISO/IEC 17025:2017 standard [27] is a valuable tool for laboratories to confirm that their data are accurate and reliable. This document guides the laboratory to develop a procedure to record data for detecting and monitoring trends. To achieve this objective, the laboratory should use reference and quality control materials, perform regular inspections of the equipment’s performance, execute replicate testing and retests, and use intermediate checks to verify consistency in results. As was already mentioned, ensuring the validity of results is required for laboratories accredited under ISO/IEC 17025, but the concepts can be applied to any laboratory performing testing. On top of this, the crucial point is that using a set of predefined criteria for the results of the analysis of data provides a means for evaluating generated data to ensure that it is of a quality that is fit for purpose. Taking action on results that are found to be outside of the predefined criteria allows the laboratory to take action to correct any issues and ensure that any nonvalid results are not reported.

This paper aims to draw attention to the importance of ensuring the validity of results, whether it is routine or in research laboratories. Comparing the results of research laboratories and verifying scientific hypotheses also require attention to the quality of the results. In this context, the recommendations of the ISO/IEC 17025 standard provide a good guideline for preparing a scenario for the process of supervising the performance of the measuring system. It is particularly significant in proteomics, where a large number of parameters affect the final result. Therefore, we have proposed a description of all of the steps necessary to ensure the proper preparation of the LC–HRMS system for proteomic measurement. This paper outlines a guideline and standardized procedure for LC–HRMS system preparation for proteomic applications, which is straightforward and should be easy to follow.

## 2. Results

**Figure 1:**
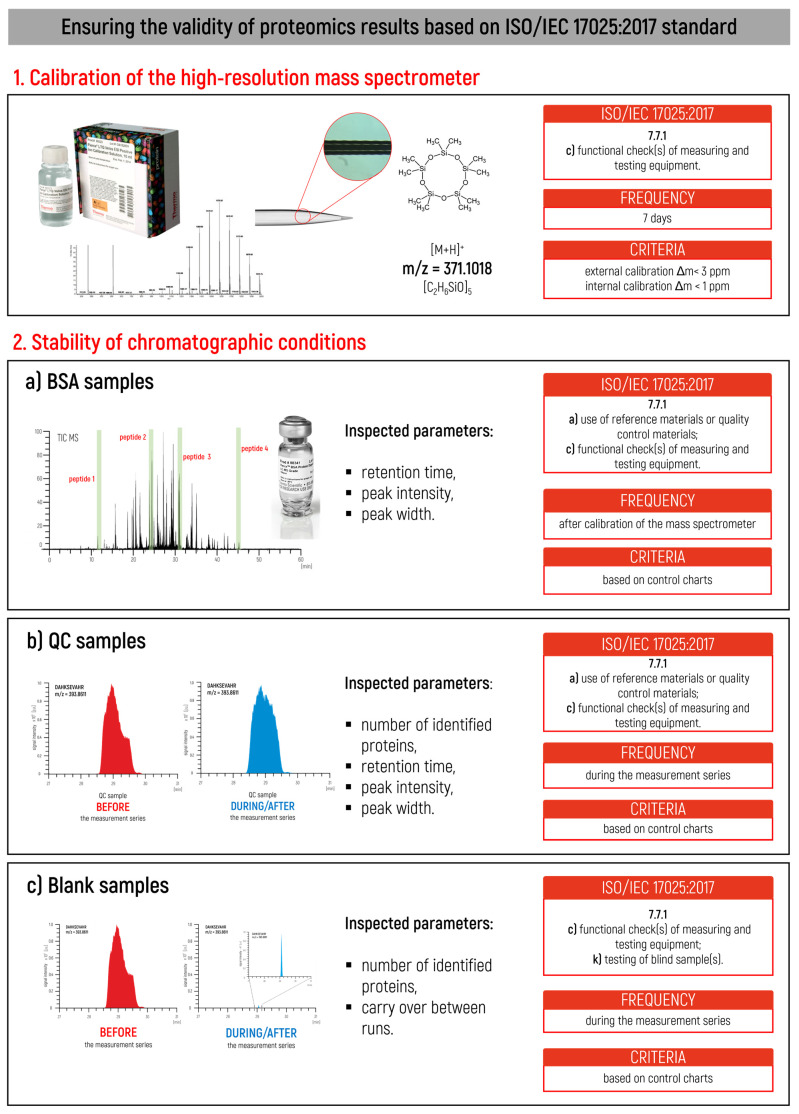
Main steps in ensuring the validity of proteomics results based on the ISO/IEC 17025:2017 standard.

This section describes in detail the rules related to ensuring the validity of proteomics results in accordance with the ISO/IEC 17025:2017 standard [27]. The most critical steps, including the criteria and recommended frequency, are shown in Figure 1. The standardized guideline approach we have presented allows for the monitoring and continuous inspection of instrumental parameters during proteomic measurements. The proposed requirements based on our experience describe the realized requirement of the ISO/IEC 17025:2017 standard [27], as well as the required frequency and criteria for the tested parameters. The guideline is divided into two stages: (1) calibration of the high-resolution mass spectrometer and (2) stability of chromatographic conditions. The first stage critically describes the measurement parameters involved in calibrating a high-resolution mass spectrometer with an Orbitrap mass analyzer before routine measurements proceed, while the second stage is concerned with monitoring crucial chromatographic parameters before and during the implementation of proteomic analysis with simple and complex control samples. Those parameters that are important to follow in both stages and are vital in obtaining valid results from the proteomic analysis are summarized further in the section.

### 2.1. Calibration of the High-Resolution Mass Spectrometer

Mass accuracy is an essential value for the significant interpretation of the mass spectra of compounds of interest in a wide range of biochemical research. To obtain accurate mass (m/z) measurements, mass spectrometers require calibration using ions of known m/z. The appropriate selection of the calibration mixture of interest should be adjusted to the type of mass spectrometer and the purpose of the analysis. The preparation of the calibration mixture, the ionization efficiency of the reagent, and memory effects should also be taken into consideration [35]. The right calibrants for LC–ESI–MS should [37] (1) be free from memory effects, (2) not cause source contamination through the introduction of nonvolatile material, and (3) be applicable in both positive- and negative-ion mode. The m/z range of the calibration mixture used is expected to cover the full m/z measurement window. The type of analyte is also important to consider, both mass and charge. In the case of the analysis of compounds characterized by high mass and multicharged ions, the use of substances of similar structure is appropriate for calibration. Calibration of a mass spectrometer is most often accomplished by the use of vendor-provided calibration mixtures. In the case of Orbitrap-based mass spectrometers, Pierce^TM^ LTQ ESI Positive Ion Calibration Solution is used. The calibration mixture consists of caffeine (20 μg/mL), MRFA (1 μg/mL), and Ultramark 1621 (0.001%) in an aqueous solution of acetonitrile (50%), methanol (25%) and acetic acid (1%). In our laboratory, calibration is performed at an interval of 7 days based on criteria elaborated and recommended by the spectrometer manufacturer: 3 ppm of Δ mass for external calibration and 1 ppm of Δ mass for internal calibration. External calibration of the mass spectrometer includes several parameters, which also comprise the calibration of adequate voltages of mass analyzers and ion-routing multipoles. Although the calibration is very frequent, according to our experience the drift of the instrument can be noticeable and the calibration needs to be reperformed. Spectrometer drift can be caused by the contamination of the system and changes in the environmental conditions of the laboratory (humidity and temperature). This problem can become more noticeable in the application of high-resolution mass analyzers than low-resolution analyzers, which are more stable over time and require less frequent recalibration [35]. To verify the stability of the received calibration, we monitor mass accuracy measurement for one selected m/z in postcalibration spectra between intervals to ensure the proper measuring conditions. For this purpose, we observe the m/z of polysiloxane ([C_2_H_6_SiO]_5_; m/z = 371.1018), one of the common background contamination ions in MS derived from the glass emitter in the ion source.

In 1984, Sack et al. [31] proposed a five-step process to confirm the calibration conditions, including accuracy and the precision of the mass measurements in routine operations. Despite significant technological advances in the development of mass spectrometers, these criteria remain continuously valid. The criteria proposed by Sack et al. [31] to ensure the reliable results of mass measurements are as follows:

1) Sample a statistically significant number of mass spectra to allow valid statistical interpretation of the results.
2) Cover a wide mass range, or at least the mass range of interest.
3) Sample ions of different relative abundances to determine the effect of the number of ions on mass measurements.
4) Statistically evaluate the mass measurement accuracy and precision for individual masses along with the overall mass measurement accuracy and precision of the entire data set.
5) Statistically analyze the error distributions to aid in the search for systematic errors.

If the stability and proper functioning of the MS system are confirmed, we can proceed to the next step of ensuring the validity of the results.

### 2.2. Stability of Chromatographic Condition

Monitoring the stability of chromatographic parameters in proteomic measurements is crucial in properly identifying proteins of interest and changes in their expression whenever a comparison is required between study groups. The reference standard ISO/IEC 17025:2017 (Section 6.4 “Equipment”) [27] requires that each instrument, including a liquid chromatograph, which is a part of the measuring system for proteomic analysis, should be verified before use and possess a maintenance protocol to protect it from accidental adjustment, which can generate invalid results. In laboratories, the verification of the chromatograph and then monitoring of chromatographic conditions are usually achieved using properly prepared control samples. The selection of the control sample should be appropriate for the measurements planned concerning the type and matrixes of the analyte under investigation. The control sample should be well characterized for its properties. The control sample should be subjected to the same procedure as the analytical samples. The common commercially available control samples used in proteomics studies can be single protein digest samples such as bovine serum albumin (BSA) or of high complexity such as tryptic digests of HeLa cells, yeast, and *Escherichia coli* proteins mixed in defined proportions [30]. The first parameter that should be checked is sequence coverage between experimental and theoretical protein sequences. Peptides/proteins selected for sequence coverage monitoring should be characterized by relatively high intensities and different retention times to cover the entire measurement window by inspection. It is important to consider that the same peptide/protein may have a completely different intensity in a standard sample and a real sample due to the many physicochemical parameters on which ionization in an LC–MS/MS system depends. The next step after initial sequence coverage analysis is an accurate evaluation of the abovementioned chromatographic parameters. For this purpose, we select several chromatographic signals present on the TIC chromatogram and evaluate them individually. Each peak comes from an independent peptide ion with a given m/z value. The decision about chosen peptide ions should be well-thought-out and includes ions covering the initial, middle, and final measuring range. It is also important to analyze peptide ions with different peak properties. The acceptance criteria for the obtained parameters are developed based on previous measurements, the type of instrument of measurement, and the characteristics of the control samples used. If the criteria are not fulfilled, appropriate steps should be taken.

#### 2.2.1. Sequence Coverage

The sequence coverage parameter is the number of amino acids in a specific protein sequence that was found in the peptides sequenced in the MS/MS study. Analysis of the fragmentation spectra obtained during measurements allows us to determine the amino acid sequence of the analyzed protein mixture. Using dedicated software containing information about protein sequences and comparing the experimentally obtained fragmentation spectra with theoretical spectra, we can determine the sequence coverage.

#### 2.2.2. Retention Time

Retention time is the time elapsed between sample introduction (beginning of the chromatogram) and the maximum signal of the given compound at the detector, which means the amount of time that the given peptide is retained on the column after it has been injected. As an example, for the bovine serum albumin sample containing several different peptides (Figure 1(2a)), each compound in the sample will retain for a different amount of time on the column according to its chemical composition, i.e., each will have a different retention time.

#### 2.2.3. Peak Width and Peak Intensity

Peak width depends on the mass resolution. A typical definition of unit resolution is when the peak width at half height is about 0.6 to 0.8 mass units. The peak width of a chromatographic peak is the peak’s full width at half maximum. Lower peak widths indicate better chromatographic resolution; an increase in width means broader LC peaks. It is worth pointing out that an abrupt change in the peak width from the previous run may indicate bleeding or tailing of the LC peaks caused by a dirty or warn-out column and that a gradual increase in width over weeks may mean that the LC column needs to be replaced. The peak intensity, defined as the intensity of the chromatographic signal, is also instantaneous in monitoring the measurement conditions, which enables the monitoring of system stability.

The control results for the key parameters during proteomic measurement are shown in Table 1. The results were recorded for measurements of a control sample containing the BSA protein. The results for two different peptides m/z = 582.3223, and m/z = 653.3630 are shown in Table 1.

### 2.3. Mass Spectrometry Analysis of Complex Proteomic Samples

In the case of the mass spectrometry analysis of complex proteomic samples, peptide separation and MS/MS identification are more challenging. Our experience demonstrates that using a single protein (BSA) as a control sample in many complex analyses might not be sufficient. This is a major problem in quantitative proteomic studies without the use of isotopic labeling [5,6], where direct information about protein concentration is obtained from the intensity of the chromatographic signal. In such cases, it is necessary to ensure that each of the several chromatographic runs in the measurement series was performed under the same conditions.

An efficient sample separation method has to be coupled with the best peptide separation system to characterize as many unique peptides and identify as many proteins in a given lysate as possible. To achieve maximum efficiency in identifying and quantifying proteins, the separation efficiency of the chromatography system and the detection speed of the mass spectrometer must match in resolution and speed, respectively. Efficient chromatography allows not only for better separation of the analytes but also improves ionization. To achieve accurate quantitation, the concentration of each protein has to be measured both accurately and reproducibly, which requires a well-established and highly validated MS method.

To develop a mass spectrometry method before the analysis of high-complexity protein samples, proteomics laboratories most often use the HeLa Protein Digest Standard [38,39]. The HeLa Protein Digest Standard is a highly validated mammalian protein digest that can be used as a quality control (QC) sample for liquid chromatography separation, MS method development, and MS performance benchmarking. HeLa S3 cells express over 15,000 proteins with relevant post-translational modifications, making this cell line an ideal standard for complex proteome mass spectrometry applications.

**Figure 2:**
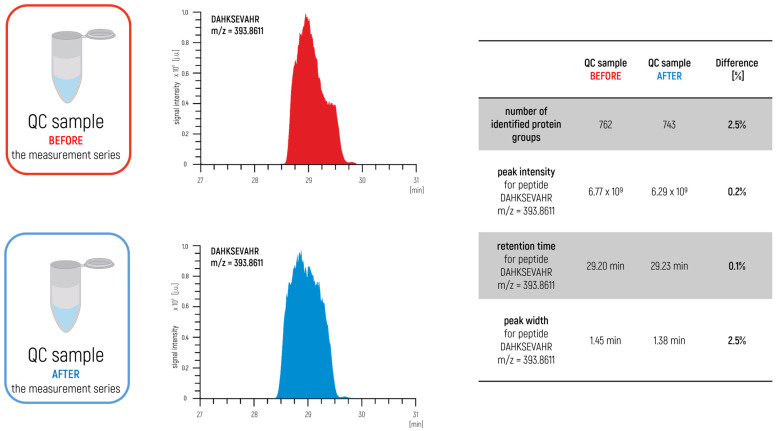
Monitoring the stability of the LC–MS/MS system using a control sample obtained after mixing all of the samples of the measurement series (XIC chromatograms for peptide DAHKSEVAHR m/z = 393.8611 and comparison of selected parameters).

The approach proposed in our latest papers [5,6] is also an interesting concept for preparing a relevant complex control sample for proteomics studies. In the case of quantitative analysis, a control sample was prepared for testing the stability of the measurement system by mixing an appropriate portion of all samples from the measurement series into a single complex control sample (Figure 2).

**Figure 3:**
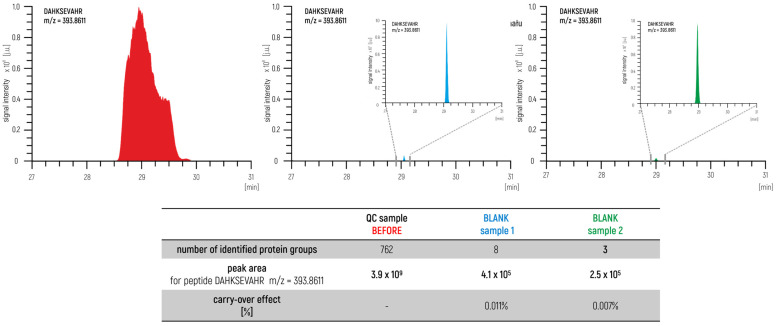
Monitoring the carry-over effect in an LC–MS/MS system using a control sample and a blank sample (XIC chromatograms for peptide DAHKSEVAHR m/z = 393.8611 and comparison of selected parameters).

In this way, the prepared sample represented an excellent degree of the qualitative and quantitative composition of the test group. The sample was measured before and after the measurement series. In this approach, we also measured blank samples to control the carry-over effect (Figure 3) to identify a potential problem within a measurement system such as the overloading of samples or problems with the proper maintenance of the LC–MS/MS. The agreement of the achieved parameters is used as a confirmation to ensure the validity of the obtained results.

### 2.4. Other Crucial Parameters of the Measurement System in High-Resolution Mass Spectrometry

Besides the parameters described above that are relevant to the operation of the measurement system in proteomics research, other additional parameters are worth analyzing as well. Significant advances in instrument software also allow us to monitor those parameters that can affect the number of identified proteins and further biological interpretation of the results. In the event of instability in the operation of the LC–MS system, information on these parameters can prove to be very useful and will enable the early reaction to return the system to the correct state.

#### 2.4.1. Spray Stability

This parameter relates to the stability of ionization in time. It is the singular most important parameter because it ensures the proper creation of ions and in that sense determines the performance of the mass spectrometer and the outcome of the calibration. Spray stability can be affected by the flow speed of the calibrant solution, which is being applied using a syringe pump. Additional parameters influencing spray stability are auxiliary and sheath gas flows, which need to be optimized to ensure the best possible spray stability. The stability of the spray is measured as a relative standard deviation (RSD, %) from a moving average of total ion chromatogram intensity. Ideal spray stability would be an RSD of around 2%. In most cases, this is not achievable; nonetheless, RSD should not exceed 5%. The software-provided threshold value, above which spray is considered to be unstable, is 15% RSD.

#### 2.4.2. Transmission

This parameter relates to the ratio of ions, which reach the detector, to the number of ions generated in the ion source. The large surface area detector (LSAD) is located in the ion trap. The ideal value for this parameter would be 100%, which means that all of the generated ions reach the detector, which is unfeasible in a real-life situation. Ion loss is an inevitable fact of mass spectrometric measurements, especially in the case of a mass spectrometer with nonlinear ion optics. In our experience, the transmission of 60–70% is satisfactory. One thing worth mentioning is that ion transmission decreases in time as the number of measured samples increases. It is worth evaluating this parameter and recording its values. When transmission drops below 50%, cleaning of the ion optics, including the S-lens, quadrupole, and ion-routing multipole, is advised. As one can imagine, the lower the transmission through the quadrupole, the lower the number of ions measured in the Orbitrap, which can lead to unsatisfactory and lower-than-expected numbers of peptide spectral matches, peptide IDs, and, in turn, protein IDs.

#### 2.4.3. Orbitrap Resolution

This parameter relates to the ability of a mass analyzer to separate ions with relatively close m/z ratios. Resolution in mass spectrometry is calculated as the ratio of the m/z value of a particular peak and its width at 50% of the peak height (FWHM). Quadrupole resolution is important, since during the measurement, it functions as an ion filter, so the proper separation of ions based on their m/z ratio is crucial. In the case of the Orbitrap mass analyzer, where the distinction between ions with m/z ratios identical up to the third decimal place is necessary, proper calibration of their resolution is needed. During proteomic measurements, an Orbitrap resolution depends on the instrument model, however, it is important to consider MS2 spectra acquisition with a lower resolution than MS1.

#### 2.4.4. Fourier Transform

Fourier transform is a mathematical transform that decomposes functions depending on space or time into functions depending on spatial or temporal frequency. In the case of Orbitrap, the Fourier transform is used for decomposing frequency measured by the Orbitrap for each ion into the m/z value of a particular ion, which allows for the acquisition of mass spectra. Its proper calibration is important because, without it, the acquisition of mass spectra is not feasible, even if the mass analyzer is functioning properly as such, i.e., it is acquiring proper frequency values for each ion.

#### 2.4.5. AGC Target Prediction

The active gain control (AGC) target refers to the cumulative number of ions accumulated in the C-trap, which are to be injected into the Orbitrap mass analyzer. When using Orbitrap, each measurement requires the accumulation of a certain number of ions in the C-trap. This amount is specified within the measurement method. The calibration of the AGC target is important, as it is a pivotal parameter that allows for proper proteomic analysis. This parameter can have an enormous impact on the final results, as it controls the actual amounts of ions that are collected in the C-trap and that in turn can be analyzed in the Orbitrap. If the AGC target prediction has not been checked and is not properly tuned, this might result in unsatisfactory numbers of peptide spectral matches and peptide and protein identifications. In advanced spectrometers, parameters such as ‘Fourier transform’ and ‘AGC target prediction’ are calibrated automatically according to the relevant frequency, but their significance must be considered during measurements.

### 2.5. Control Tools for Monitoring Analytical Parameters

**Figure 4:**
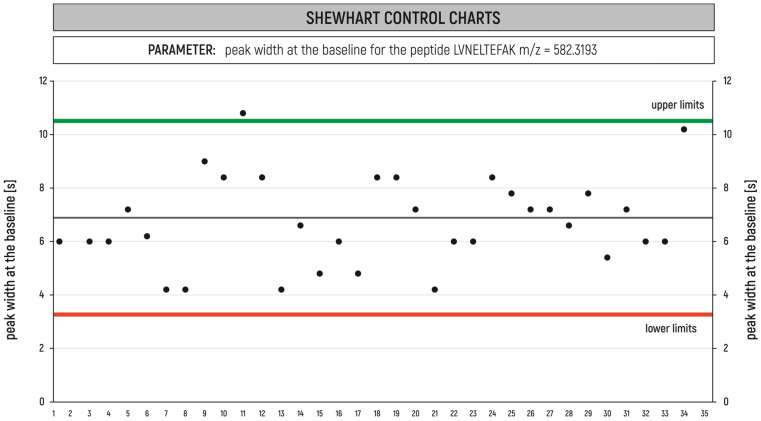
Shewhart charts for peak width at the baseline for the selected peptide LVNELTEFAK m/z = 582.3193 from the BSA control sample.

The statistical quality control technique is used to determine the control limits and for exploring the changes that require improving the process. In our laboratory, we most frequently use the Shewhart control chart for quality control of the results. The Shewhart control chart has a baseline and upper and lower limits, shown as dashed lines, that are symmetrical about the baseline. Measurements are plotted on the chart versus a timeline. The measurements that are outside of the limits are considered to be out of control. In proteomics studies, the Shewhart control charts are successfully applied to check the monitoring of valid proteomics results. An example of Shewhart charts for peak width at the baseline for the selected peptide LVNELTEFAK m/z = 582.3193 from the BSA control sample is shown in Figure 4.

## 3. Discussion

In the case of proteomic analysis, the obtained results are used in many areas, including in the evaluation of protein expression under drug administration, the identification of novel biomarkers in neurodegenerative disorders, the metabolism of psychotropic substances, or the investigation of plant physiology and metabolism. Thus, there is a big expectation to obtain valid results, as they could be used to conclude the status of organisms.

The requirements of ISO/IEC 17025 and ISO 15189 are widely used in accredited laboratories. Unfortunately, quality control issues are often omitted in scientific papers, making it impossible to compare published results and thus draw general conclusions. For the use of advanced mass spectrometry techniques in routine applications such as medical diagnostics, as well as in many regulated areas, ensuring the validity of the reported results and consistency of analytical performance is crucial.

The use of complex protein mixtures during the calibration process would adversely affect the quality and resolution of individual signals. Simple calibration mixtures with a low number of peptides provide very high-resolution peaks from the individual peptides, allowing more detailed analysis. Moreover, the use of complex control solutions increases the probability of peptide co-elution, which impedes the process of data analysis. It is worth mentioning that calibration with a simple peptide mixture greatly facilitates perception and adjustment of errors that occurred during the measurement, e.g., it is exceptionally challenging to notice a significant loss in the number of identified proteins/peptides using calibration solutions rich in proteins/peptides, which may attest to the leakiness of the nano-liquid chromatography system. Due to the numerous advantages of simple calibration mixtures, proteomic laboratories frequently use peptide mixes obtained after the enzymatic hydrolysis of bovine serum albumin (BSA) as a control sample to monitor chromatographic parameters. Another interesting approach is to prepare a control sample that represents the quantitative and qualitative composition of all samples from the measurement series. The analysis of such a control sample allows the simple evaluation of chromatographic parameters, i.e., retention time, peak intensity, peak width, carry-over effect, and their comparison data obtained during previous calibrations and data suggested by the manufacturer. Additionally, the proper calibration of all of the necessary parameters of the high-resolution mass spectrometer is of utmost importance. This paper, as an example, uses the Orbitrap mass analyzer-based mass spectrometer, which is often used in the field of proteomics. Aside from the most well-known parameters such as spray stability and mass analyzer resolution, it is worth mentioning lesser-known parameters that also need to be carefully monitored, since they have an impact on the results of the analysis, i.e., quadrupole transmission and automated gain control target adjustment and monitoring.

## 4. Materials and Methods

### 4.1. Reagents

Analytical grade chemicals and analytical standards were obtained from Merck (Darmstadt, Germany), Promega (Madison, WI, USA), Thermo Scientific (Bartlesville, OK, USA), and EMD Millipore (Darmstadt, Germany). Deionized water obtained from the Milli-Q system (18.2 MΩ cm; EMD Millipore, Darmstadt, Germany) was used for samples and standard dilution.

### 4.2. Instrumentation

The instrumentation used for sample preparation was as follows: mechanical homogenizer Ultra-Turrax (IKA, Königswinter, Germany), laboratory incubator CLN 240 (MultiSerw, Brzeźnica, Poland), vacuum centrifuge 5804/5804 R (Eppendorf, Enfield, CT, USA), vortex shaker (IKA, Königswinter, Germany), thermomixer Eppendorf Comfort (Eppendorf, Enfield, CT, USA), and vacuum concentrator SpeedVac Concentrator Plus (Eppendorf, Enfield, CT, USA). Reversed-phase capillary nano-UHPLC separations were performed using an UltiMate 3000 nanosystem (Dionex Ultimate Series UHPLC, Thermo Scientific, Enfield, CT, USA) coupled online with a high-resolution tandem mass spectrometer (Orbitrap Fusion Tribrid™ Mass Spectrometer, Thermo Scientific, Enfield, CT, USA).

### 4.3. LC–MS/MS Analysis

Liquid chromatography–tandem mass spectrometry analysis was performed using an Orbitrap Fusion^TM^ Tribrid^TM^ mass spectrometer (Thermo Fisher Scientific, Enfield, CT, USA) coupled online to a nanoflow UHPLC instrument (Ultimate 3000 UHPLC, Thermo Fisher Scientific, Enfield, CT, USA). One microgram of enzymatically-cleaved peptides was separated over a 52 min gradient on a reverse-phase 50 cm long C18 in-house packed column (75 μm inner diameter, ReproSil Gold 120 C18, 1.9 μm beads (Dr. Maisch, Ammerbuch, Germany)). Solvent A consisted of 0.1% formic acid in water; solvent B consisted of 0.1% formic acid in 80% acetonitrile. The eluted peptides were ionized in the positive ion mode in the nano-ESI source with a capillary voltage of 1.9 kV. Mass spectrometric analysis was performed in data-dependent acquisition mode, with dynamic exclusion set to 60 s. Survey scans (350–1600 m/z, target value 1 × 10^5^, and maximum ion injection times 30 ms) were acquired from the Orbitrap mass analyzer at a resolving power of 60,000. MS/MS spectra were acquired from the Orbitrap mass analyzer in the top-speed mode for the most abundant, multiply-charged ions. Precursor ions with an intensity above 1 × 10^6^ were chosen for higher-energy collisional dissociation (HCD)-based fragmentation (normalized collision energy 30). The MS/MS scans were acquired at a resolving power of 15,000 (target value 5 × 10^4^ and maximum ion injection times 60 ms).

### 4.4. Data Analysis

Raw MS data were converted to mgf files using MSConvert (v. 3.0.18294-6c5e69c23) software. All MS/MS data were searched using Mascot (Matrix Science, London, UK). The following parameters were set: trypsin was set as a digestion enzyme; precursor and fragment ion mass tolerances were 5 ppm and 10 ppm, respectively; carbamidomethylation on cysteine was a fixed modification; carbamylation on lysine and N-terminus were variable modifications; up to two missed cleavage sites were allowed. Proteins and peptides (minimum six amino acids) were identified using the target-decoy approach with the reversed database. The results were processed with a false discovery rate set to 0.01 at the level of both peptides and proteins.

## 5. Conclusions

In this work, we drew attention to the need to develop a unified protocol for assuring the quality of proteomic analysis results. The purpose of the presented protocol was the need to ensure the validity of the results in studies that are often related to medical research. The developed detailed standardized guideline includes all essential items enabling ensure the validity of the results in proteomic studies based on international standards such as ISO/IEC 17025 and ISO 15189. The presented scenario demonstrates the calibration of the high-resolution mass spectrometer, monitoring of the stability of chromatographic conditions, and suitable tools for analyzing trends in changes during the monitoring of parameters. Undoubtedly, standardized procedures impress obligations on laboratories working in the regime of quality systems, in accordance with the relevant standards. On the other hand, academic laboratories are not subject to such strictly formalized procedures; researchers can choose the most optimal measurement conditions for their aims. Nevertheless, the purpose of this paper was to draw attention to the fact that in scientific research, the quality of results is equally as important. Comparing results, and thus verifying hypotheses requires at least a good and detailed description of the experimental conditions and proper supervision of the stability of the measurement system. In our view, the proposed scenario fulfills both the requirements of routine testing as well as research laboratories, assuring confidence in the validity of the results. This guideline is intended to be used by quality managers and analytical staff in both industry and academia who are involved in the planning, performance, and management of nonroutine measurements in analytical science and associated research and development.

## Figures and Tables

**Table 1:** Examples of inspected key parameters for proteomic measurement (laboratory data of BSA control sample).

Data of Measurements	Key Parameters for Proteomic Measurement						
		m/z = 582.3223			m/z = 653.3630		
	Sequence Coverage (%)	Peak Width	Retention Time	Signal Intensity	Peak Width	Retention Time	Signal Intensity
day 1	77%	6.0	34.97	3.36 × 10^9^	7.2	31.79	4.47 × 10^9^
day 2	78%	6.0	35.31	2.25 × 10^9^	7.2	31.94	3.24 × 10^9^
day 3	70%	6.0	36.81	1.67 × 10^9^	5.4	33.77	1.31 × 10^9^
day 4	77%	7.2	36.87	5.44 × 10^9^	7.2	33.83	3.47 × 10^9^
day 5	67%	7.3	42.54	5.96 × 10^9^	5.4	40.03	1.09 × 10^9^
day 6	60%	6.3	37.3	2.44 × 10^9^	4.2	34.36	1.53 × 10^9^
day 7	74%	6.5	37.33	2.11 × 10^9^	7.3	34.42	1.24 × 10^9^
day 8	89%	9.0	37.28	7.40 × 10^9^	7.2	34.28	6.91 × 10^9^
day 9	87%	8.4	37.24	6.19 × 10^9^	8.4	34.26	4.15 × 10^9^
day 10	90%	8.7	37.16	5.94 × 10^9^	9.6	34.19	3.57 × 10^9^
day 11	87%	8.4	37.1	4.80 × 10^9^	10.8	34.11	2.16 × 10^9^
day 12	74%	6.5	37.12	2.24 × 10^9^	4.8	34.18	1.76 × 10^9^
day 13	60%	6.6	37.29	1.85 × 10^9^	5.4	34.25	1.32 × 10^9^
day 14	80%	4.8	37.22	1.22 × 10^9^	6.0	34.22	6.77 × 10^8^
day 15	74%	6.0	37.12	1.37 × 10^9^	6.0	34.14	6.14 × 10^8^
day 16	76%	4.8	36.85	2.48 × 10^9^	6.0	33.85	1.31 × 10^9^
day 17	78%	8.4	36.17	1.58 × 10^9^	8.4	33.06	1.56 × 10^9^
day 18	68%	8.4	36.19	1.66 × 10^9^	8.4	33.06	1.37 × 10^9^
day 19	67%	7.2	36.11	7.57 × 10^8^	6.0	33.03	1.23 × 10^9^
day 20	68%	5.3	39.38	7.20 × 10^8^	6.0	36.32	1.04 × 10^9^
day 21	68%	6.0	39.62	8.91 × 10^8^	6.1	36.58	1.67 × 10^9^
day 22	66%	6.0	39.68	9.06 × 10^8^	6.0	36.63	1.37 × 10^9^
day 23	67%	8.4	37.19	6.50 × 10^8^	6.0	34.16	2.70 × 10^8^
day 24	76%	7.8	37.51	1.20 × 10^9^	6.0	34.33	1.25 × 10^9^
day 25	78%	7.2	37.41	1.25 × 10^9^	7.2	34.3	9.49 × 10^8^
day 26	75%	7.2	37.75	1.59 × 10^9^	7.2	34.62	1.03 × 10^9^
day 27	72%	6.6	38.98	9.68 × 10^8^	9.6	35.9	8.99 × 10^8^
day 28	71%	7.8	38.98	1.28 × 10^9^	6.0	35.86	2.39 × 10^9^
day 29	67%	5.4	39.64	2.08 × 10^9^	6.6	36.46	1.93 × 10^9^
day 30	71%	7.2	39.58	1.49 × 10^9^	6.6	36.44	2.01 × 10^9^
day 31	80%	6.0	39.95	1.35 × 10^9^	5.4	36.74	1.74 × 10^9^
day 32	76%	6.0	39.91	1.18 × 10^9^	7.2	36.72	1.93 × 10^9^
day 33	89%	8.1	40.98	9.19 × 10^8^	6.5	38.03	1.03 × 10^9^
day 34	80%	7.5	40.85	6.24 × 10^8^	7.1	37.99	7.86 × 10^8^
Average Value	75%	6.91	37.98	2.29 × 10^9^	6.78	34.96	1.86 × 10^9^
min	60%	4.80	34.97	6.24 × 10^8^	4.20	31.79	2.70 × 10^8^
max	90%	9.00	42.54	7.40 × 10^9^	10.80	40.03	6.91 × 10^9^

## Data Availability

Not applicable.
